# RETRACTED: Salem et al. Prostate Cancer Metastases Are Strongly Inhibited by Agonistic Epha2 Ligands in an Orthotopic Mouse Model. *Cancers* 2020, *12*, 2854

**DOI:** 10.3390/cancers18182964

**Published:** 2026-09-14

**Authors:** Ahmed F. Salem, Luca Gambini, Sandrine Billet, Yu Sun, Hiromichi Oshiro, Ming Zhao, Robert M. Hoffman, Neil A. Bhowmick, Maurizio Pellecchia

**Affiliations:** 1 Division of Biomedical Sciences, School of Medicine, University of California Riverside, 900 University Avenue, Riverside, CA 92521, USA; afs.abdalla@gmail.com (A.F.S.); lucaga@ucr.edu (L.G.); 2 Department of Medicine, Cedars-Sinai Medical Center, 8700 Beverly Boulevard, Los Angeles, CA 90048, USA; sandrine.billet@cshs.org (S.B.); neil.bhowmick@cshs.org (N.A.B.); 3 AntiCancer Inc., 7917 Ostrow St., San Diego, CA 92111, USA; sy831225@gmail.com (Y.S.); hiromichi-oshiro@hotmail.com (H.O.); mzhao@anticancer.com (M.Z.);; 4 Department of Surgery, University of California, San Diego, CA 92037, USA

The journal retracts the article titled “Prostate Cancer Metastases Are Strongly Inhibited by Agonistic Epha2 Ligands in an Orthotopic Mouse Model” [1], cited above.

Following publication, concerns were brought to the attention of the Editorial Office regarding the integrity of figures published in this article [1].

In accordance with the journal’s standard procedures, the Editorial Office and Editorial Board conducted an investigation that identified indications of inappropriate editing and duplication in panels presented in Figure 1, including material overlapping with a previously published article [2] produced by a different authorship group. Although the authors cooperated with the investigation, the explanations and supporting materials provided were not deemed sufficient by the Editorial Board to resolve the identified concerns. As a result, the Editorial Board has lost confidence in the reliability of the findings and decided to retract this publication [1], as per MDPI’s retraction policy.

This retraction was approved by the Editor-in-Chief of the journal *Cancers*.

The authors have been informed of this retraction and have indicated their disagreement with this decision. Dr. Bhowmick takes responsibility for the preparation of the data relative to Figure 1, and the associated methods section and figure caption.
